# Should I drink water or wine? Impacts of online nutritional counseling on the alcohol consumption of elderly individuals with mild cognitive impairment: Secondary Data Analysis from the RCT “BrainFit-Nutrition”

**DOI:** 10.1186/s12889-026-29728-5

**Published:** 2026-10-03

**Authors:** Julia-Sophia Scheuermann, Elmar Graessel, Petra Scheerbaum, Etienne Hanslian, Julia Katharina Schiele, Christian S. Kessler, Carolin Donath

**Affiliations:** 1 Section of Medical Psychology and Medical Sociology, Department of Psychiatry and Psychotherapy, Uniklinikum Erlangen, Friedrich-Alexander-Universität Erlangen-Nürnberg (FAU), Schwabachanlage 6, Erlangen, 91054 Germany; 2 Center for Health Services Research in Medicine, Department of Psychiatry and Psychotherapy, Uniklinikum Erlangen, Friedrich-Alexander-Universität Erlangen-Nürnberg (FAU), Schwabachanlage 6, Erlangen, 91054 Germany; 3 Department for Dementia Research and Nursing Science, University for Continuing Education Krems, Krems, Austria; 4 Department of Internal Medicine and Nature-Based Therapies, Immanuel Hospital Berlin, Berlin, 14109 Germany; 5 Charité Competence Center for Traditional and Integrative Medicine (CCCTIM), Charité - University Medical Center Berlin, Corporate Member of Freie Universität Berlin and Humboldt-Universität Zu, Berlin, Germany; 6 Department of Integrative Health Care and Prevention, University of Augsburg & University Hospital Augsburg, Augsburg, Germany

**Keywords:** Alcohol drinking, Cognitive dysfunction, Distance counseling, Elder nutritional physiological phenomena

## Abstract

**Background:**

People with mild cognitive impairment (MCI) have an increased health risk when they consume alcohol in comparison with people without cognitive impairment. Excessive alcohol consumption is an established modifiable risk factor that contributes to the risk of developing dementia.

**Objective:**

Within a secondary data analysis of a 2 × 2 randomized controlled trial (RCT) – consisting of a computerized cognitive intervention and a nutrition counseling intervention –, we evaluated the impact of a nutritional counseling group (neither focusing solely on alcohol consumption) on alcohol consumption in people with MCI at a 12-months follow-up, identified predictors of changes in alcohol consumption, and determined whether women benefited in the same way as men.

**Methods:**

One hundred eighty-nine (189) of 270 trial participants reported consuming alcohol at baseline and started one of the two nutritional counseling programs (ITT sample). Data were collected at a 12-months follow-up and imputed for *n* = 15 participants. Participants received either whole-food plant-based (WFPB) nutritional group counseling or nutritional group counseling based on the German Nutrition Society’s (DGE) recommendations. Alcohol consumption was quantified with standard drinks (10 g alcohol)/day. Multiple linear regressions were used to identify potential predictors of reduction. *P*-values were corrected.

**Results:**

The sample’s average age was 70.6 years and nearly showed gender parity (52% female). Both interventions led to a significant reduction in alcohol consumption in people with MCI after 12 months (Cohen''''s *d* = .30 (WFPB); Cohen''''s *d* = .39 (DGE)). There were no significant differences in the change in consumption level over time between the nutritional counseling groups (*p* = .173). Women substantially reduced their alcohol consumption over time only in the DGE nutritional counseling group (Cohen''''s *d* = .33). In successful alcohol reducers (*n* = 84), higher baseline alcohol consumption (β = .670, *p* < .001), older age (β = .158, *p* = .035), and stricter adherence in the counseling group (β = .184, *p* = .017) were significant predictors of a greater reduction (*F*(7,76) = 18.539, *p* = .003). Higher cognitive functioning was a significant negative predictor of reduction (β = -.199, *p* = .009).

**Conclusion:**

In context of the lifestyle intervention of the BrainFit-Nutrition study, nutritional counseling for people with MCI was effective for reducing alcohol intake. Our results suggest focusing on younger people living with MCI as well as higher educated people, especially men, to optimize the potential of modifiable risk factors for dementia. However, gender differences exist. A gender-specific nutritional counseling program is advised in the light of the gender health gap.

**Trial registration:**

ISRCTN10560738; registered 11/23/2021.

**Supplementary Information:**

The online version contains supplementary material available at https://doi.org/10.1186/s12889-026-29728-5.

## Background

Due to changes in demographics and living conditions, the number of people with dementia is increasing both in Germany and worldwide [1, 2] with an even larger increase in low- and middle-income countries [3]. This increase is accompanied by an increase in the prodromal stage of dementia with age: mild cognitive impairment (MCI) [4]. According to Livingston et al. [5], there are 14 modifiable risk factors that influence the development of dementia across the lifespan. In Germany, it is estimated that 36% of all cases of dementia are related to modifiable risk factors [6]. Thus, a 15–30% reduction in the prevalence of these modifiable risk factors could reduce the number of dementia cases by 330,000 cases over the next few years. Excessive alcohol consumption, defined as 21 british alcohol units (168 g pure alcohol) per week, is one of those 14 modifiable risk factors [5], particularly prevalent in mid-life, when excessive alcohol consumption is statistically at its highest [7, 8].

Germany has one of the highest alcohol consumption levels in the European Union [9, 10]. In addition to factors such as lack of physical exercise and depression, excessive alcohol consumption also contributes to a reduction in cognitive reserve and causes neuropathological damage [5]. Increased alcohol consumption is therefore also a risk factor for the development of MCI and cognitive decline [11–13]. There is a non-linear dose–response relationship between alcohol consumption and the progression to dementia with an exponential increase in the risk of cognitive decline starting at 27.5 g/day [11]. Thus, not only consumption but also the amount consumed appear to be relevant factors that influence cognitive decline. Research to date has provided contradictory results [12, 14], as it shows both (potential, but questionable) benefits and increasing risk depending on the level of consumption [15–18]. In some studies, no influence of alcohol consumption on the risk of conversion from MCI to dementia has been shown [11, 19]. However, this does not mean that alcohol has no harmful effects. The reported protective influence of low alcohol consumption on the MCI-dementia-conversion rate [20–22] should be viewed critically and with caution, because, due to the study design, so-called “sick quitters”—individuals who had already reduced their consumption due to health limitations—may have been included in the control groups or non-drinker groups [23]. Alcohol consumption is a neurotoxic substance because toxic byproducts are produced during the metabolic process. Therefore, there is no protective effect associated with low alcohol consumption, nor is there any level of consumption that is risk-free for health [13, 24, 25]. Moderate alcohol consumption causes long-term atrophy of the hippocampus and, as a result, impaired cognitive function [13, 14, 26]. However, people with cognitive impairment tend to report, following the sick quitter effect, lower alcohol consumption than people without cognitive impairment [17, 22] at least in Asian and American populations, but there is gender-dependent higher consumption in Western populations favoring women [27]. Nevertheless, a consistent positive relationship was found between alcohol consumption and the conversion rate [11, 19, 20].

Women and men show several physiological differences, including a slower alcohol metabolism in women, thus leading to higher blood alcohol levels with comparable amounts of alcohol in women than in men [28, 29]. However, recent findings show that even the smallest amount of consumption has negative consequences for both genders [24, 30]. Gender differences in alcohol consumption are due not only to biological factors but also to social factors [31]. Men consume more alcohol than women [29, 32], with a gender gap primarily in older age cohorts. However, the gender gap is narrowing due to an increase in alcohol consumption among women at least in high-income countries. With increased alcohol consumption, women are more susceptible to direct alcohol-associated impairments (including cognition) and even more vulnerable than men to the negative societal consequences of harmful consumption (e.g., involuntary sexual intercourse, sexually transmitted diseases because of unprotected sexual interactions, unplanned pregnancies). It is therefore particularly important to contribute further research on the gender health gap in the area of substance consumption.

The effects of unspecified nutritional counseling on the alcohol consumption of older people living with MCI have not yet been directly investigated. However, such counseling is expected to have a positive effect on alcohol consumption. Nutritional interventions may directly or indirectly influence alcohol consumption levels (depending on its content) and consequently improve general health and cognitive functioning [33, 34]. Nutritional advice is also part of a more comprehensive lifestyle intervention. Counseling often integrates long-term strategies to maintain a balanced diet and healthy lifestyle. Dietary approaches such as the Mediterranean or MIND diets (Mediterranean-DASH Intervention for Neurodegenerative Delay), focusing on foods that are beneficial for brain health, have been associated with less cognitive decline and could potentially influence habits such as alcohol consumption [34]. For this purpose, the effects of two non-pharmacological intervention arms on the cognitive performance of individuals living with MCI were investigated by means of a randomized controlled trial (RCT) *BrainFit-Nutrition* (BF-N) [35]—namely, a cognitive intervention and nutritional intervention. The nutritional part of the intervention included neuroprotective, plant-based nutritional counseling training for the intervention group and nutritional counseling training based on the recommendations of the German Nutrition Society (DGE – Deutsche Gesellschaft für Ernährung) for the active control group of the RCT.

Thus, in the present secondary data analysis, we a) examined the potential influence of two nutritional counseling groups intervention (unspecific concerning consumption behavior) on changes in alcohol consumption in people living with MCI after 12 months. In addition, we b) investigated which predictors were associated with a sustainable reduction in alcohol consumption. Furthermore, because of the existing gender differences in harmful alcohol consumption of the sample at study baseline [27] and gender-specific risk parameters of alcohol consumption, we c) examined gender-specific differences in the intervention effects. All analyses of the effects of nutritional counseling were conducted within the context that these were part of a two-arm RCT.

We posed three research questions in this secondary data-analysis:

1. Does a nutritional counseling intervention (unspecific concerning alcohol consumption behavior) during an 2 × 2 non-pharmacological intervention change alcohol consumption in the long-term in consumers living with mild cognitive impairment?
2. Are there gender-specific differences in the efficacy of the nutritional counseling intervention concerning the level of alcohol consumption after the nutritional intervention?
3. What are substantial predictors of a sustainable reduction in alcohol consumption in individuals with mild cognitive impairment?

## Methods

### Study design and sample

**Fig. 1:**
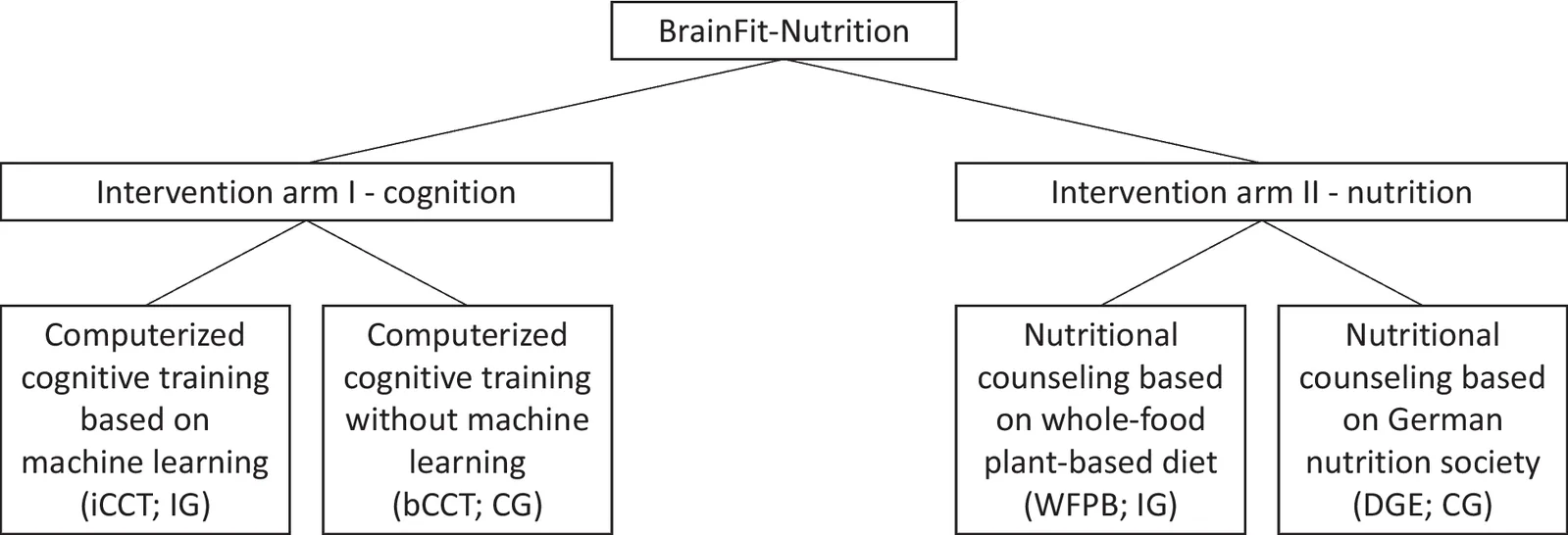
RCT arms of the BrainFit-Nutrition study. *Notes*. IG = intervention group; CG = Control group. The current analysis focused on intervention arm II

We analyzed data from the RCT BF-N. The study design of the prospective RCT included a 2 × 2 design with two intervention arms (see Fig. 1), (I) computerized cognitive training (CCT) and (II) online nutritional counseling. For both arms, an active intervention specialized for people with MCI and an active control measure was developed. The combination of the two intervention arms with two active control conditions resulted in four active groups to which the study participants living with MCI were randomly assigned, stratified by age, gender, and Montreal Cognitive Assessment (MoCA) baseline score.

Therefore, all participants received during the RCT a combination of one CCT and one nutritional counseling. Only the type of counseling and cognitive training varied across the four possible combinations (see Fig. 1). The study was conducted entirely digitally, as both interventions were carried out digitally and the regular assessments were also conducted entirely digitally. The study design has already been published [35] and registered in ISRCTN (ISRCTN10560738). The primary hypothesis of the trial concerned cognition as the primary outcome. The outcome of this paper (alcohol consumption) was not included in the primary analysis.

**Fig. 2:**
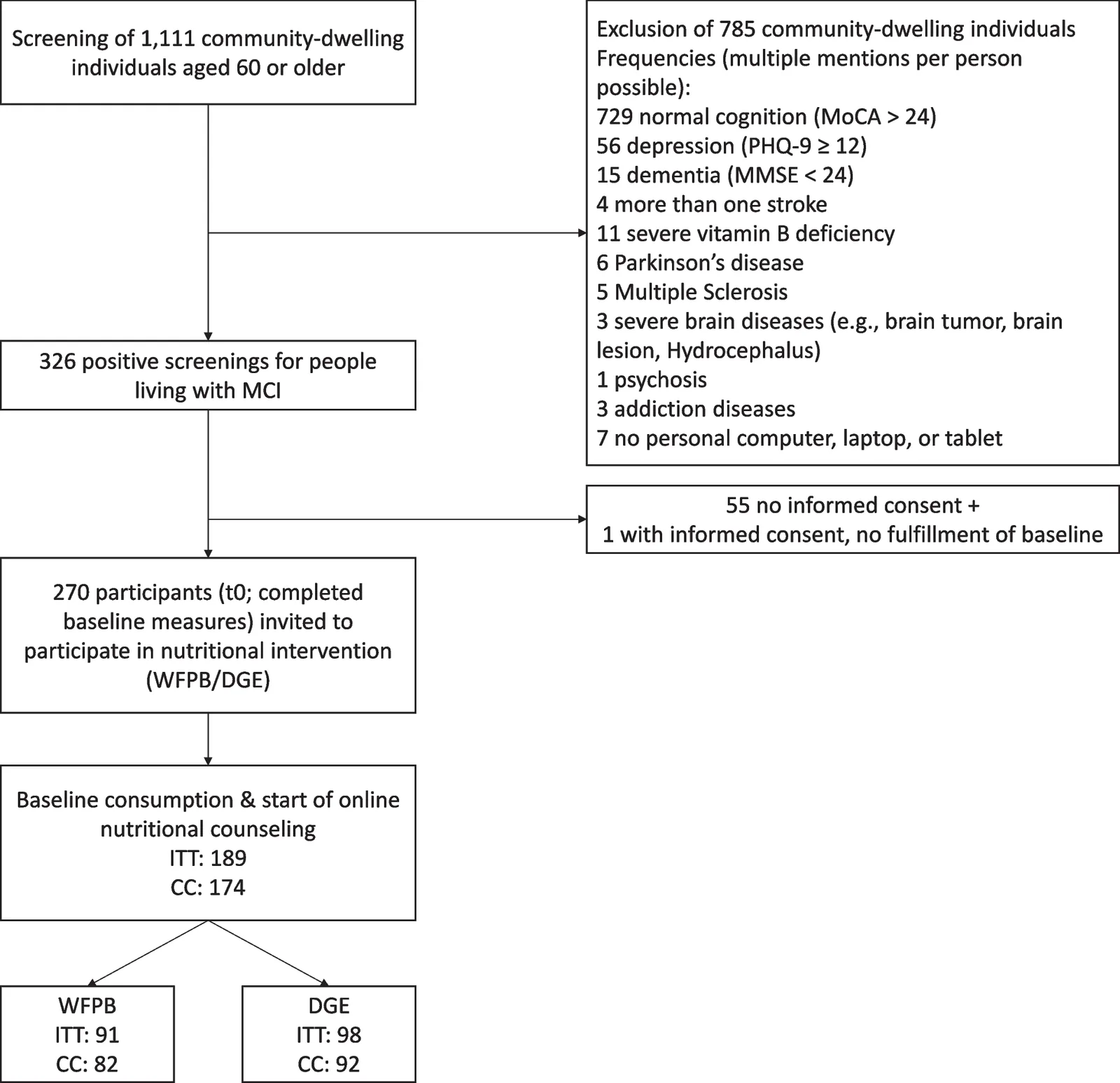
Participant flowchart. *Notes*. MoCA: Montreal Cognitive Assessment; PHQ-9: Patient-Health Questionnaire-9; MMSE: Mini-Mental-Status Examination; WFPB: whole-food plant-based intervention; DGE: German Nutrition society; ITT: intention-to-treat; CC: complete case

In the BF-N study, 1,111 people aged 60 and older in Germany were screened for MCI between January and September 2022 [35]. To recruit participants, numerous recruitment approaches were attempted all over Germany, such as (company) health insurance funds, newspaper announcements, social media, flyers, advertisements on public transportation etc. The participants were people with cognitive impairment living in their homes, not being institutionalized. Participants were living either alone or with a (family) caregiver. The primary inclusion criterion for participation was a MoCA score ≤ 24 points and a Mini-Mental Status Examination score ≥ 24 points, which identified 326 individuals living with MCI. Of these, 271 individuals living with MCI agreed to participate and were randomized. A total of *N* = 270 people completed the baseline survey with a telephone interview (see Fig. 2). In the present analysis, those who had reported alcohol consumption at baseline and started the nutritional intervention (participation in at least one counseling session) were included (*n* = 189). In the complete case (CC) analyses, only participants who took part in the 12-months follow-up were considered (*n*_*CC*_ = 174). For intention-to-treat (ITT) analyses, alcohol consumption was imputed for *n* = 15 (*n*_*ITT*_ = 189) participants. For the strategy, see the statistical analysis.

All procedures were approved by the ethics committee of the medical faculty of the Friedrich-Alexander-Universität Erlangen-Nürnberg (Ref.: 21-318_1-B). A prospective registration of the RCT was conducted in the ISRCTN Registry (ISRCTN10560738).

### Interventions: online nutritional counseling

The BF-N study comprised—besides a computerized cognitive training program—two kinds of online nutritional counseling. Each nutritional counseling course included 12 manualized online group sessions, each lasting 90 min in a biweekly mode.

Neither course focused specifically on alcohol consumption. The issue of alcohol consumption was addressed in both groups as part of a session on oils, fats, nuts, seeds, and drinks. Both recommended, first, no consumption of any alcohol at all. If alcohol was to be consumed at all, then it was advised to do so in moderation and to consume preferably red wine [36]. In both nutritional intervention groups, the neurotoxic effects of alcohol and the negative effects of consumption on the performance and capacity of the brain were pointed out. Due to the minor thematic differences with regard to alcohol consumption and its consequences between the two groups, we aimed to explore the effects of a general online nutritional counseling (two varieties neither focusing on alcohol consumption behavior) in this study, which were combined with a CCT intervention.

#### Counseling focusing on whole-food plant-based nutrition (WFPB)

The whole-food plant-based (WFPB) nutrition intervention was based on anti-inflammatory and neuroprotective components [35]. The recommended maximum tolerable alcohol consumption was 10 g/day for healthy women and 20 g/day for healthy men. The participants were given information on the alcohol concentration of the various alcoholic drinks and the calories they contained as well as the recommendation to abstain from alcohol. They were informed of the neurotoxic and pro-inflammatory effects of alcohol as well as the increase in existing symptoms such as tiredness and weakness caused by alcohol.

#### Counseling focusing on recommendations of the German Nutrition Society (DGE)

This group received nutritional counseling based on the recommendations of the DGE, which recommends an omnivorous diet. The participants were not informed about the grams and calories of the individual alcoholic drinks; otherwise, the content of the counseling on alcohol consumption was identical to that of the WFPB group. Thus, participants were informed about the neurotoxic effects of alcohol and the negative effects on brain capacity and performance. They were given the recommendation not to consume any alcohol.

### Instruments

Cognitive performance was assessed with the MoCA screening instrument [37], range 0–30 points. The original test recommends a cutoff score of 26 on the MoCA for MCI [37]. However this cutoff does not yield optimal results. Instead, the literature now even favors a cutoff of < 24 points [38–40]. According to Thomann et al. [38], the sensitivity–specificity curve intersects at approximately 24 points, which is why a cutoff of ≤ 24 was chosen in the present RCT study.

Depressiveness was evaluated using the PHQ-9 scale [41], range 0–27 points. The sociodemographic data recorded were age, gender (female/male), employment status (still working yes/no), living situation (living alone, yes/no), educational level (based on the International Classification of Education, ISCED: low, middle, high [42]), and body mass index (BMI). The adherence to nutritional counseling was recorded as the number of sessions attended.

Alcohol consumption behavior was recorded during the interview using adapted items from the ESA survey (IFT) [43] and the GEDA study [7]. The 12-months prevalence (“How often did you drink alcohol in the past 12 months?”; 5-point scale ‘never’ to ‘daily’) and 30-day prevalence for alcohol consumption were recorded, as well as the number of days of consumption in the last month (“How many of the past 30 days have you consumed alcohol?”; number), the number of alcoholic SDDs per day (“How much alcohol do you usually drink if you consume alcohol?”; 10 g pure alcohol = 1 SDD), and the number of days with five or more alcoholic drinks in the last 30 days (“How many days in the past 30 days have you had 5 or more alcoholic drinks on a single occasion (e.g., in one evening)?”, number of days).

### Statistical analysis

According to the CONSORT checklist, the main analyses reported here are ITT analyses with all participants of the BF-N study who were consumers at baseline, started the nutritional intervention, and completed the six-months follow-up (t6) survey. A conservative approach was chosen to impute missing values from individuals who only participated in the follow-up survey at t6 but not at the one-year follow-up (t12). Thus, t12 values were imputed by the “Last-Observation-Carried-Forward” (LOCF) method with the corresponding t6 values. To examine potential differences in baseline characteristics between t12 responders and non-responders, t-tests and χ^2^ tests were conducted. As sensitivity analyses, CC analyses were also conducted with all participants in the BF-N study who completed the t12 follow-up. The analysis strategies for ITT and CC are described in detail below for addressing research questions 1 to 3. The analyses were calculated with SPSS V.31.0.0.0. The tests were two-sided and the α-error level was set to 0.05. A correction of the α-error was carried out as specified by Benjamini and Hochberg [44]. Only the corrected *p*-values are reported in the manuscript.

#### Descriptive analysis

As part of the descriptive analysis, we calculated the arithmetic mean (*M*) and standard deviation (*SD*) or the frequency and percentage (%) of the sociodemographic variables, alcohol consumption at baseline and t12, and the change in alcohol consumption (difference t12-t0).

#### Research question 1

Research question 1 was analyzed with the subsample of consumers at baseline who at least started the nutritional intervention (*n*_*ITT*_ = 189; *n*_*CC*_ = 174).

##### Bivariate analysis

Using t-tests for dependent samples, the change in consumption behavior over time (t12-t0) was examined within the two nutritional groups. Group differences between the two nutritional counseling groups were analyzed using t-tests for independent samples (dependent variable: change in the alcohol consumption level t12-t0). Assumptions for t-tests were tested: box plot graph for outliers, Shapiro–Wilk test for a normal distribution, Levene’s test for homogeneity of variance.

##### Multivariate analysis

Multivariate testing of group differences was performed using multiple linear regression with alcohol consumption at t12 as the dependent variable. In preparation for the multivariate analysis, bivariate correlations were computed as Pearson/Spearman correlations or η coefficients depending on the scaling of the variables (significance *p* < 0.05). Baseline alcohol consumption level, adherence (sum score session participation), nutritional counseling group (WFPB (1) vs. DGE (0)), age, gender (male (0)/female (1)), and education (dichotomized ISCED classification: high education (1) vs. low education (0)) were included in the model as fixed predictors. The assumptions for linear regression were tested before: (I) Linearity: graphically using a scatterplot; (II) Outlier: analysis of studentized excluded residuals, leverage values with cut-off values according to Igo [45] and Vellemann and Welsch [46] and use of “Cook’s Distance”; (III) Independence of residuals: Durbin-Watson statistics; (IV) Multicollinearity: tolerance values and variance inflation factor.

#### Research question 2

The sample used to address research question 2 was based on the sample analyzed for research question 1, but the analyses were performed specifically for each gender with t-tests for a) dependent and b) independent variables to evaluate descriptive differences concerning the alcohol consumption level at t12 a) within each group and b) between the nutrition counseling groups. A multiple linear regression was computed to evaluate predictors of consumption levels after the intervention. The ITT sample consisted of 98 women (*n*_W-WFPB_ = 45, *n*_W-DGE_ = 53) and 91 men (*n*_M-WFPB_ = 46, *n*_M-DGE_ = 45); the CC analysis comprised 91 women (*n*_W-WFPB_ = 40, *n*_W-DGE_ = 51) and 83 men (*n*_M-WFPB_ = 42, *n*_M-DGE_ = 41).

#### Research question 3

For research question 3, only the baseline consumers who had actually reduced their alcohol consumption at t12 (and at least started nutritional counseling) were considered (*n*_*ITT*_ = 84). Multiple linear regression was used to identify predictors associated with a sustainable reduction in alcohol consumption in individuals living with MCI (dependent variable: reduction amount t0-t12). The multiple linear regression procedure is described above, and assumptions were tested. The following variables were included: adherence (sum score session participation), baseline alcohol consumption, nutritional counseling group (WFPB (1)/DGE (0)), age, gender (male (0)/female (1)), education (dichotomized ISCED classification: high education (1) vs. low education (0)), depressiveness (PHQ-9; [41]), living situation (solely (0)/shared household (1)), frequent smoker (no (0)/yes (1)), and Body Mass Index (BMI).

## Results

### Sample characteristics

The ITT sample (*n*_*ITT*_ = 189) comprised 48.1% (*n* = 91) of the participants from the WFPB group and 51.9% (*n* = 98) of the participants from the DGE group. About half of the sample were women, and the majority of participants were highly educated and retired. On average, they participated in 8 out of 12 nutritional counseling sessions and reduced their daily alcohol intake between baseline and the 12-months follow-up by ⅓ alcoholic SDD. Table 1 presents the descriptive characteristics.

**Table 1:** Sample characteristics of the ITT sample (*N* = 189)

**Variable**	**Total (*N* = 189)** *M*(*SD*) or *n*(%)	**Female (*n* = 98)** *M*(*SD*) or *n*(%)	**Male (*n* = 91)** *M*(*SD*) or *n*(%)
Age, *M*(*SD*)	70.64 (6.68)	69.91 (0.68)	71.43 (0.69)
Range	[60;86]	[60;85]	[60;86]
IQR	10.5	9	9
Sex (female), *n*(%)	98 (51.9%)	-	-
Education, *n*(%)			
High	114 (60.3%)	41 (41.8%)	73 (80.2%)
Medium	68 (36.0%)	51 (52.0%)	17 (18.7%)
Low	7 (3.7%)	6 (6.1%)	1 (1.1%)
BMI, *M*(*SD*)	25.55 (4.20)	24.88 (4.14)	26.27 (4.16)
Employment (yes), *n* (%)	55 (29.1%)	32 (32.7%)	23 (25.3%)
Adherence (number of nutritional counseling sessions), *M*(*SD*)	7.89 (3.27)	7.53 (3.44)	8.27 (3.04)
Living situation (shared household), *n* (%)	132 (69.8%)	55 (56.1%)	77 (84.6%)
Depressiveness (PHQ-9), *M*(*SD*)	3.74 (2.9)	4.51 (3.1)	2.90 (2.4)
Cognition			
MoCA t0, *M*(*SD*)	22.37 (1.57)	22.74 (1.33)	21.96 (1.7)
Range t0	[16;24]	[19;24]	[16;24]
MoCA t12, *M*(*SD*)	24.32 (2.73)	24.68 (2.8)	23.92 (2.62)
Range t12	[16;29]	[16;29]	[16;28]
Alcohol consumption^+^ (standard drinks with 10 g or 12 g alcohol/day), *M*(*SD*)			
t0	1.61 (0.95)	1.5 (0.82)	1.73 (1.06)
Range t0	[0.125;5.0]	[0.125;5.0]	[0.125;5.0]
t12	1.28 (0.83)	1.28 (0.8)	1.28 (0.86)
Range t12	[0.0;5.0]	[0.0;5.0]	[0.0;4.0]

*Notes. * Education according to ISCED [42]; *BMI* Body Mass Index, *PHQ-9* Patient Health Questionnaire-9, *MoCA *Montreal Cognitive Assessment, *t0* Baseline, *t12* 12-months follow-up, ^+^only consumers at t0

An analysis of the t12 non-responders (*n* = 15) revealed that there were no significant differences in baseline sociodemographic characteristics compared to the t12 responders (*n* = 174), with the exception of adherence to dietary counseling and BMI: the responders attended significantly more sessions than the non-responders (Responder: *M* = 8.18, *SD* = 3.11; Non-Responder: *M* = 4.53, *SD* = 3.23; *t*(187) = −4.34, *p* < 0.001), but also a higher BMI than the non-responders (Responder: *M* = 25.70, *SD* = 4.28; Non-Responder: *M* = 23.78, *SD* = 2.42; *t*(187) = −1.71, *p* = 0.044).

### Research question 1

#### Main analysis – ITT

##### Changes in alcohol consumption level over time

**Fig. 3:**
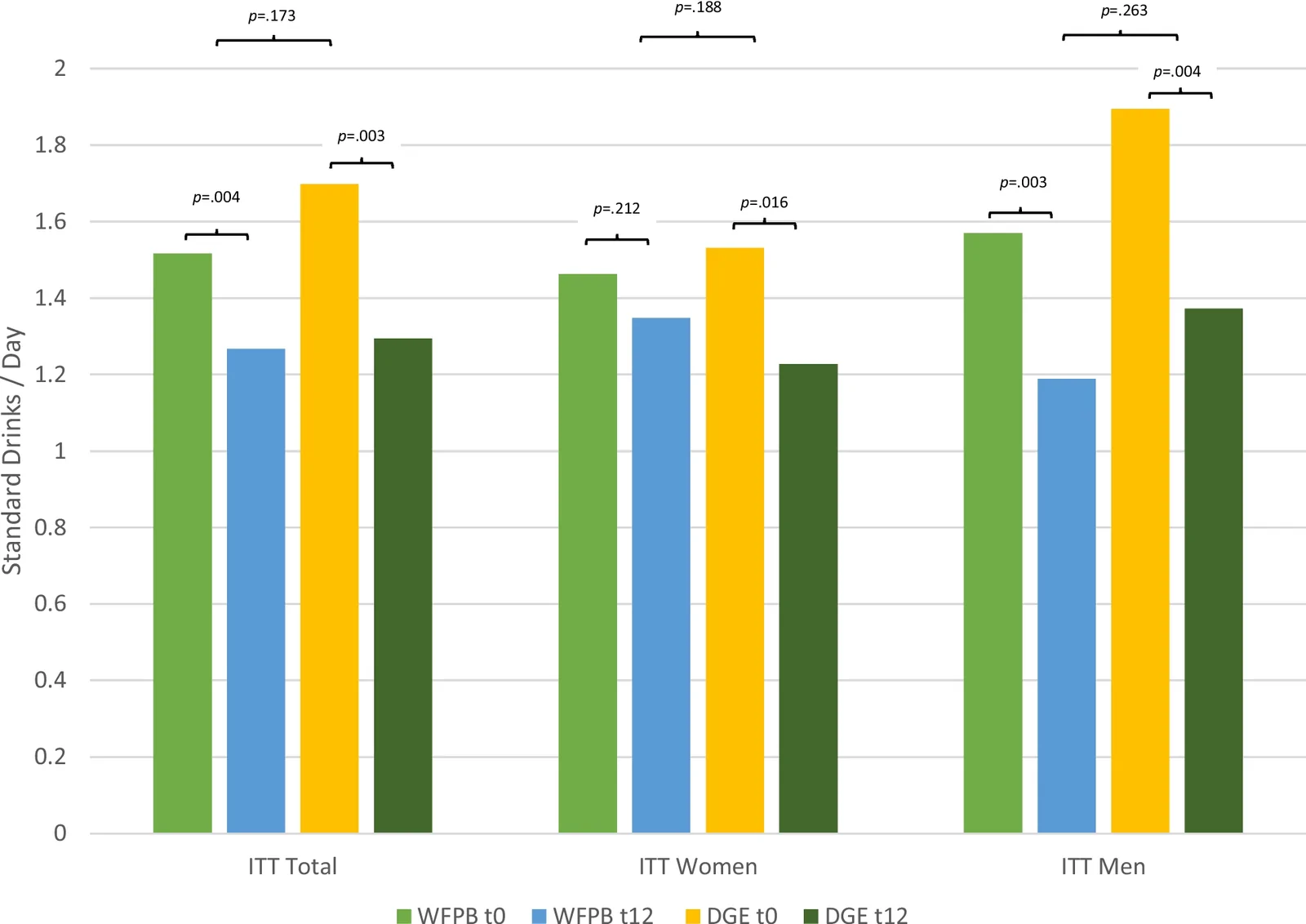
Group differences between WFPB and DGE nutritional counseling**.***Notes*. ITT: intention to treat; WFPB: whole-food plant-based; DGE: German Nutrition Society

In the WFPB group, participants (*n* = 91) reduced their average daily alcohol intake by approximately ¼ SDD: at t12 (*M* = 1.27, *SD* = 0.79), alcohol consumption was significantly lower than at t0 (*M* = 1.52, *SD* = 0.78), *t*(90) = 2.85, *p* = 0.004, Cohen''''s *d* = 0.30. The assumptions for the t-test were met. Also in the DGE counseling group (*n* = 98), participants significantly lowered their daily consumption by an average of ⅖ SDD: At t12 (*M* = 1.29, *SD* = 0.86), alcohol consumption was significantly lower than at t0 (*M* = 1.70, *SD* = 1.08),* t*(97) = 3.84, *p* = 0.003, Cohen''''s *d* = 0.39. The assumptions for the t-test were met. Figure 3 illustrates the extents to which each group reduced their consumption.

##### Group differences between WFPB and DGE nutritional counseling

Even though, descriptively, participants reduced their alcohol intake in the DGE group to a greater extent than in the WFPB group, there was no significant difference between the WFPB (*M* = −0.25, *SD* = 0.84) and DGE groups (*M* = −0.40, *SD* = 1.04; *t*(183.1) = −1.130, *p* = 0.173, Fig. 3). The assumptions for the t-test were met.

##### Multivariate analysis

Multiple linear regression was performed to explore the association of group (WFPB vs. DGE counseling) with the t12 alcohol consumption level while controlling for other variables. In the bivariate pre-analysis, we detected significant correlations between the t12 alcohol consumption level and a) the baseline consumption level (*r* = 0.434, *p* < 0.001) and b) age (*r* = −0.188, *p* = 0.010).

The check for linear regression assumptions resulted in the exclusion of three outliers because at least two of the criteria for outliers were met. All other prerequisites for multiple linear regression were met. The multiple linear regression analysis resulted in a significant model, *F*(6, 179) = 7.444, *p* = 0.003, with one significant predictor of alcohol consumption at t12: t0 alcohol consumption (β = 0.396, *p* < 0.001) (Table 2), such that higher baseline alcohol consumption was associated with higher alcohol consumption 12 months later. Furthermore, education (β = 0.145, *p* = 0.051) and age (β = −0.120, *p* = 0.084) showed a statistical trend, indicating that higher education might be associated with higher consumption level at t12 and MCI persons in the younger old group at study start appeared to show higher consumption at t12. In the inferential statistical analysis, the group variable (kind of nutritional counseling) was not significantly associated with consumption at t12. The amount of explained variance (*R*^*2*^ = 0.200) was moderate [47].

**Table 2:** Multiple linear regression ITT sample (*n* = 186)

Variable	*B*	*SE*	β	*t*	*p*	95% *CI* (*B*)
Constant	1.631	.606		2.692	**.008**	[.435;2.826]
Adherence	−0.019	.016	-.081	−1.191	.235	[-.050;.012]
Group^§^ (WFPB)	0.046	.103	.031	.448	.655	[-.158;.251]
Gender ^~^ (female)	0.084	.114	.055	.736	.463	[-.141;.309]
Education°	0.224	.114	.145	1.968	*.051*	[-.001;.449]
Age	−0.014	.008	-.120	−1.738	.*084*	[-.029;.002]
Baseline alcohol consumption	0.332	.059	.396	5.656	**<.001**	[.216;.447]

*Notes. *^§^Nutritional counseling group (WFPB (1) vs. DGE (0)), ~ gender (male (0)/female (1)); °education was dichotomized based on ISCED classification (high education (1) vs. low education (0)). *WFPB* Whole-food plant-based, *DGE* German Nutrition Society, *B* unstandardized regression coefficient, *SE* Standard error of unstandardized regression coefficient *B*, *β* Standardized regression coefficient, *t t*-Test, *p* Significance, significant values are bold, statistical trends in italics, *95%-CI* Confidence interval of the unstandardized coefficient

#### Sensitivity analysis – complete case analysis

The CC sample comprised *N*_*CC*_ = 174 individuals, with *n* = 82 participants in the WFPB group and *n* = 92 in the DGE group. The descriptive characteristics are shown in additional material 1. Alcoholic drinks per day were reduced by about ⅓ SDD (*M* = −0.35, *SD* = 0.97) from t0 (*M* = 1.62, *SD* = 0.97) to t12 (*M* = 1.27, *SD* = 0.84).

For the CC sample, the results of the ITT analysis were essentially supported: Both nutritional groups showed significant reductions in alcohol intake (*p* = 0.004, Cohen''''s *d* = 0.33 for WFPB and *p* = 0.003, Cohen''''s *d* = 0.39 for DGE). There was no significant difference in change in alcohol consumption between the two groups (*p* = 0.212). The multivariate analysis revealed a significant model, as in the ITT sample, *F*(7,163) = 5.762, *p* = 0.003, with moderate amount of explained variance (*R*^*2*^ = 0.198). Baseline alcohol consumption was also the only significant variable (β = 0.389, *p* < 0.001). A detailed description of the results for the CC sample and the gender-specific analysis for the CC sample is reported in additional material 2.

### Research question 2

#### Female ITT sample

##### Changes in the alcohol consumption level over time

Within the female WFPB group (*n* = 45), there was no significant difference between the t12 (*M* = 1.35, *SD* = 0.75) and t0 consumption levels (*M* = 1.46, *SD* = 0.81), *t*(44) = 0.91, *p* = 0.212, Cohen’s *d* = 0.14. The reduction amounted ⅒ of an SDD. The assumptions for the t-test were met. By contrast, the female DGE group (*n* = 53) reduced their consumption by about ⅓ SDD and therefore showed a significantly lower alcohol consumption level at t12 (*M* = 1.23, *SD* = 0.84) than at t0 (*M* = 1.53, *SD* = 0.84), *t*(52) = 2.39, *p* = 0.016, Cohen''''s *d* = 0.33. The assumptions for the t-test were met.

##### Group differences between WFPB and DGE nutritional counseling

The decrease in the female DGE group was slightly larger than in the female WFPB group. Nevertheless, women in the WFPB (*M* = −0.11, *SD* = 0.85) and the DGE (*M* = −0.30, *SD* = 0.93) groups showed no significant difference in how much their alcohol consumption decreased (*t*(96) = −1.04, *p* = 0.188, Fig. 3). The assumptions for the t-test were met.

##### Multivariate analysis

The correlation-based bivariate pre-analysis showed a significant bivariate correlation between the t12 alcohol consumption level and the baseline consumption level in females (*r* = 0.392, *p* = 0.002) as well as between the t12 alcohol consumption level and age (*r* = −0.214, *p* = 0.002). With the exception of the assumption involving (II) “Outliers,” all prerequisites for the analysis were fulfilled. As a result, we excluded two outliers. The multiple linear regression analysis to evaluate predictors for the consumption behavior after the nutritional intervention at t12 within the female subsample (Table 3) resulted in a significant model, *F*(5,90) = 3.187, *p* = 0.018, and explained a medium amount of the variance of 15% (*R*^*2*^ = 0.150) [47]. One significant predictor emerged: alcohol consumption at baseline (before counseling) (β = 0.293, *p* = 0.004). The more alcohol women consumed at baseline, the more they consumed at t12. The kind of nutritional counseling (group variable) as well as the educational level was not significantly associated with the t12 consumption level.

**Table 3:** Multiple linear regression female ITT sample (*n* = 96)

Variable	*B*	*SE*	β	*t*	*p*	95% *CI* (*B*)
Constant	2.018	.803		2.514	**.014**	[.424;3.613]
Adherence	-.022	.020	-.111	−1.125	.264	[-.062;.017]
Group^§^ (WFPB)	.165	.139	.118	1.190	.237	[-.111;.441]
Education°	.010	.138	.007	.071	.943	[-.265;.285]
Age	-.016	.010	-.152	−1.515	.133	[-.037;.005]
Baseline alcohol consumption	.287	.099	.293	2.915	**.004**	[.091;.483]

*Notes. *^§^Nutrition counseling group (WFPB (1)/DGE (0)). *WFPB* Whole-food plant-based, *DGE* German Nutrition Society. °Education was dichotomized based on ISCED classification (high education (1) vs. low education (0)). *B* Unstandardized regression coefficient, *SE* Standard error of unstandardized regression coefficient *B*, *β* standardized regression coefficient, *t t*-Test, *p* Significance, significant values are bold, *95%-CI* confidence interval of the unstandardized coefficient

#### Male ITT sample

##### Changes in alcohol consumption level over time

Male participants in the WFPB group (*n* = 46) showed a significant decrease of about ⅖ SDD from t0 (*M* = 1.57, *SD* = 0.75) to t12 (*M* = 1.19, *SD* = 0.83), *t*(45) = 3.19, *p* = 0.003, Cohen''''s *d* = 0.47. The assumptions for the t-test were met. Within the male DGE group (*n* = 45), the alcohol consumption level decreased by about half an SDD. Thus, the consumption level at t12 (*M* = 1.37, *SD* = 0.89) was significantly lower than at t0 (*M* = 1.89, *SD* = 1.29), *t*(44) = 3.01, *p* = 0.004, Cohen''''s *d* = 0.45. The assumptions for the t-test were met.

##### Group differences between WFPB and DGE nutritional counseling

Even though the DGE group descriptively showed a larger reduction than the WFPB group, there were no significant group differences between the WFPB (*M* = −0.38, *SD* = 0.81) and DGE (*M* = −0.52, *SD* = 1.16) groups in the reduction of alcohol consumption (*t*(78.3) = −0.67, *p* = 0.263, Fig. 3) for men. The assumptions for the t-test were met.

##### Multivariate analysis

From the preliminary bivariate correlation analysis, the following variables were included in the model for men, as they had significant bivariate correlations with t12 alcohol consumption: baseline consumption level (*r* = 0.476, *p* = 0.002), age (*r* = 0.279, *p* = 0.011), and cognitive status at baseline (*r* = 0.279, *p* = 0.009).

After exclusion of one outlier, all assumptions for the multiple linear regression were met (Table 4). Thus, the analysis revealed a statistically significant model, *F*(6,83) = 6.123, *p* = 0.003, which explained about 31% (*R*^*2*^ = 0.307) of the variance with two significant predictors: baseline alcohol consumption (β = 0.462, *p* < 0.001) and education (β = 0.212, *p* = 0.037). Thus, in males, baseline alcohol consumption was also positively associated with alcohol consumption twelve months later. Further, persons with higher education indicated higher consumption level twelve months later. Neither the nutritional counseling group nor adherence were significantly associated with the alcohol consumption level at t12.

**Table 4:** Multiple linear regression male ITT sample (*n* = 90)

Variable	*B*	*SE*	β	*t*	*p*	95% *CI* (*B*)
Constant	-.235	1.632		-.144	.886	[−3.481;3.011]
Adherence	-.027	.028	-.092	-.947	.346	[-.083;.029]
Group^§^ (WFPB)	-.016	.165	.009	.097	.923	[-.312;.344]
Age	-.006	.013	-.050	-.495	.622	[-.032;.019]
Education°	.452	.213	.212	2.126	.**037**	[.029;.875]
Baseline alcohol consumption	.373	.077	.462	4.829	**<.001**	[.219;.526]
MoCA baseline score”	.054	.053	.100	1.020	.311	[-.051;.160]

*Notes. *^§^Nutrition counseling group (WFPB (1) vs. DGE (0)); “MoCA: Montreal Cognitive Assessment. *WFPB* Whole-food plant-based, *DGE* German Nutrition Society. °Education was dichotomized based on ISCED classification (high education (1) vs. low education (0)). *B* unstandardized regression coefficient, *SE* Standard error of unstandardized regression coefficient *B*, *β* standardized regression coefficient, *t t*-Test, *p* Significance, significant values are bold; *95%-CI* confidence interval of the unstandardized coefficient

### Research question 3

The multiple linear regression model to identify predictors of a sustainable reduction was significant, *F*(7, 76) = 18.539, *p* = 0.003, with four significant variables (see Table 5). This model showed according to Cohen [47] with about 63% a high explanation of variance (*R*^2^ = 0.631). Baseline alcohol consumption (β = 0.670, *p* < 0.001), age (β = 0.158, *p* = 0.035), adherence (β = 0.184, *p* = 0.017), and baseline cognitive status in the MoCA (β = −0.199, *p* = 0.009) were significantly associated with the reduction in the amount of alcohol consumed 12 months later. Thus, the older the people living with MCI and the greater their baseline consumption, the more they reduced their alcohol consumption after nutritional counseling. Furthermore, greater adherence to the nutritional counseling program and more severe cognitive impairments (lower MoCA scores) in the study participants were associated with a larger reduction in the amount of alcohol consumed at the follow-up point. At least a statistical trend was found in the multivariate model for group membership (β = 0.149, *p* = 0.051) favoring the counseling that was based on the DGE recommendations, which was associated with a larger reduction. Education, however, did not prove to be a significant predictor of a sustainable reduction. The assumptions for multiple linear regression were met  .

**Table 5:** Multiple linear regression sustainable reducers (*n* = 84)

Variable	*B*	*SE*	β	*t*	*p*	95% *CI* (*B*)
Constant	.335	.983		.337	.737	[−1.626;2.288]
Adherence	.042	.017	.184	2.449	**.017**	[.008;.076]
Group^§^ (DGE)	.212	.107	.149	1.983	.*051*	[-.001;.425]
Gender ^~^ (female)	.027	.110	.019	.245	.807	[-.193;.247]
Age	.017	.008	.158	2.152	**.035**	[.001;.033]
Education°	.094	.108	.066	.869	.388	[-.122;.310]
Baseline alcohol consumption	.468	.052	.670	9.046	**<.001**	[.365;.571]
MoCA baseline score”	-.086	.032	-.199	−2.667	.**009**	[-.151;-.022]

*Notes. *^§^Nutrition counseling group (WFPB (0) vs. DGE (1)); ~ gender (male (0)/female (1)); “MoCA: Montreal Cognitive Assessment. *WFPB* whole-food plant-based, *DGE* German Nutrition Society. °Education was dichotomized based on ISCED classification (high education (1) vs. low education (0)). *B* unstandardized regression coefficient, *SE* Standard error of unstandardized regression coefficient *B*, *β* standardized regression coefficient, *t t*-Test, *p* Significance, significant values are bold; statistical trends in italics; *95%-CI* confidence interval of the unstandardized coefficient

## Discussion

The aim of this exploratory secondary data analysis was to examine the change in alcohol consumption levels in people with MCI who participated in online nutritional group counseling during participation in an 2 × 2 RCT. The results showed that, in the overall sample, alcohol consumption levels were substantially reduced by an average of ⅓ SDD after digital nutritional counseling (equivalent to ⅓ of shandy). There were no differences between the two nutrition counseling programs regardless of the CCT program.

However, when looking at the genders separately, women reduced notably their consumption over the period of 12 months only after participating in the DGE counseling group, whereas for men, both nutritional counseling interventions were effective for reducing consumption. That men benefited more from the nutritional interventions than women, was also reflected in larger effect sizes (men: *d* = 0.45/*d* = 0.47 vs. women: *d* = 0.33/*d* = 0.14), which illustrate the higher net drinking reduction in men of about ½ SDD on average after the intervention (vs. an average reduction of ⅓ SDD in women). This finding might be due in part to a higher level of daily baseline alcohol consumption in men (1.73 SDDs) than in women (1.5 SDDs) and thus a greater intervention potential or “attack surface” of the intervention in male participants. Apparently, the efficacy potential of the online nutritional group intervention was limited up to a certain boundary; that is, in both genders, the average daily consumption level stagnated at 1.28 SDDs 12 months after the intervention. Thus, contrary to the current DGE recommendations, the majority of study participants still consumes alcohol regularly, and thereby risk negative effects on their cognitive function and overall health [24].

Thus, the digital nutrition counseling interventions in our study were associated with a reduction in alcohol consumption. The extent of the reduction in the DGE group was comparable to the results of the review by Kaner et al. [48] (2.8 SDDs/week in the current study vs. up to 3 SDDs/week in Kaner et al.), in contrast to the WFPB group with an average reduction of 1.75 SDDs/week. The results achieved by the DGE group were even more surprising, given that, unlike in the studies included in Kaner et al., the present study did not involve a dietary intervention that specifically targeted alcohol consumption. However, we also ascertained that there was no significant change in alcohol consumption in the female subsample that participated in the WFPB group. Thus, the WFPB recommendation does not appear to have been effective (in the same manner) for all participants. It is possible that the female subsample may have already been behaving more in line with the WFPB recommendations (ceiling effect), so these recommendations did not show any measurable effectiveness compared with male participants. However, in the literature, there is already evidence of gender differences in the effectiveness of digital nutrition interventions [49]. According to the literature, men benefit more than women, as women tend to live more health-conscious lives and have greater self-control, nutritional discipline, and motivation to consume healthy foods [50]. In addition, according to self-determination theory [51], nutritional interventions lead to more significant positive changes in men’s long-term food intake and greater improvements in the metabolic profiles of men [52] in comparison with women. Nutrition counseling may have promoted the three basic needs autonomy, sense of competence, and social integration, and thereby positively influenced self-determination regarding alcohol consumption. A comparison of the two dietary groups, independent of the gender of the participants, showed no significant effect, but the effect sizes in the DGE group were larger than those in the WFPB group, suggesting that the DGE recommendations are more feasible.

The baseline consumption pattern was the most substantial predictor of subsequent alcohol consumption in the present study, regardless of the type of nutritional counseling. Thus, greater baseline consumption in either the overall sample or the gender-specific subsamples was associated with greater follow-up consumption. Nevertheless, in the group of “reducers,” greater alcohol consumption at baseline led to a larger reduction in consumption. This difference can be attributed to a possible “floor effect”, meaning that low consumers may have less or no awareness of the risks of their existing alcohol consumption. However, individuals with higher consumption in these groups are sensitized to greater health awareness through nutritional counseling and subsequently reduce their consumption, regardless of the form of nutritional counseling they receive. Thus, in line with the risk factor publication by Livingston et al. [5], intensive counseling on alcohol consumption and its potential negative consequences during early middle age appears to be advisable.

Furthermore, older age predicted a larger reduction among the “reducers”. The baseline analysis [27] already suggested that elderly adults living with MCI still being younger tend to show risky alcohol consumption. Since the effectiveness of digital nutrition interventions is independent of age, and older people tend to benefit even more [49], the younger age group of elderly living with MCI appears to be “risk population” for higher consumption levels. In addition, studies have shown that increasing age is associated with a decrease in consumption frequency and amount [53–55]. However, this finding has been shown primarily in the group of “reducers”. As a practical implication this finding suggests that rather younger elderly living with MCI should be targeted in order to minimize their alcohol consumption level and the associated negative health effects.

Along with age, a greater degree of cognitive impairment also appears to be a notable factor for a larger reduction in alcohol consumption among the “reducers” in our sample. This is in line with Topiwala and colleagues, who show that cognitive decline may be a factor that goes along with a lowered alcohol consumption [13]. However, this factor—in accordance with the “sick quitter” effect—suggests that individuals likely adopt more health-conscious behaviors and therefore reduce their alcohol consumption because of the health limitations they have already experienced. This is in line with the evident increasing risk of cognitive impairment or dementia along with increased alcohol consumption [11, 19, 20, 56].

In this context, it should not be ignored that the sample was characterized by a very high level of education due to the recruitment strategy. This could have contributed to an underestimation or distortion of the correlations. Furtherly, it is possible that, in the present sample with a very high level of education, the subjective perception of memory loss may have led participants to adopt a more health-conscious lifestyle, whereby individuals with greater cognitive impairment are more likely to have reduced their consumption.

Beyond that, we could show that a high level of education according to the ISCED classification also proved to be a significant predictor for higher consumption levels at t12 especially in the male subsample. In the overall sample, this factor proved to be merely a statistical trend. Previous research shows that higher education is indeed associated with higher alcohol consumption, particularly in the moderate-to-high consumption range [30, 57]. However, it also indicates that there is most likely no direct correlation between alcohol consumption and education; rather, the amount of alcohol consumed tends to be an indicator of lifestyle, social structure, and drinking context. In line with the social causation hypothesis among adolescents and individuals in early adulthood [57], highly educated individuals drink alcohol primarily due to social and occupational contexts. In this context, education – reflecting the traditional division of roles and historical gender differences in access to higher education – emerges as a relevant factor in relation to follow-up consumption levels, particularly in the male subsample, but not in the female subsample. This indicates that, given the identification of alcohol as a relevant risk factor for the onset of dementia in middle age [5], efforts to reduce alcohol consumption should be implemented early on across all educational levels.

The frequency with which individuals participated in the nutritional counseling sessions was also a predictor of their alcohol consumption at follow-up. Individuals with MCI were able to benefit more from a comprehensive lifestyle intervention if they attended the sessions more frequently. Due to their more frequent attendance, this group of individuals, who often experienced loneliness [58, 59], met regularly in a group setting, which means that the social aspect may also have made a significant contribution to reducing their consumption. In addition, the structured format with a counselor can also contribute to greater effectiveness, especially in specific programs for problematic alcohol consumption [60].

Even though there was no statistically significant difference in the direct comparison of the two nutritional counseling programs, there are some hints in this exploratory study analysis that the training that was based on DGE recommendations has advantages:

1. Women profited only from the DGE nutritional counseling.
2. In the whole sample, participants reduced their consumption on an absolute level more in the DGE group (−0.4 SDD, equivalent to 0.1 liter of beer) than in the WFPB group (−0.25 SDD, equivalent to 0.025 liter of wine).
3. The largest absolute reduction in alcohol consumption in this study was found in a DGE group (−0.5 SDD in males, equivalent to 0.05 liter of wine or half a small glass of beer).
4. In successful reducers, participation in the DGE group predicted lower follow-up consumption at the level of a statistical trend.

### Strengths and limitations

The results are limited by the fact that this study concerns a secondary data analysis of an 2 × 2 RCT, including a combined intervention design, which can have an impact on the results. Distortions in the results due to the second intervention arm (CCT) cannot be completely excluded. Due to the secondary data analysis of the 2 × 2 RCT the sample size and consequently the group size was smaller than in a traditional survey which is usually used to examine epidemiological research questions regarding substance use. However, there is currently no register for people living with MCI and in healthy population surveys the cognitive status is not assessed. Thus, this study was a first exploratory attempt, however, future research will require further studies that examine this topic in larger samples, possibly with an untreated subsample, in order to provide reliable conclusions regarding dementia prevention. The effectiveness of both counseling programs may have been underestimated, as participants were not required to attend every nutrition counseling session. Furthermore, participants were not required to incorporate the nutritional advice into their everyday lives and neither counseling type focused specifically on alcohol consumption behavior.

In addition, the present analysis was based on a convenience sample that had a higher educational level than the general population of this age group. Thus, participants may have been more health-conscious and motivated, also due to their participation in an RCT, and thus the level of self-reported alcohol consumption might not have been comparable to that of the general population. Furthermore, biases due to social desirability with regard to self-reported alcohol consumption cannot be excluded, as no biological consumption parameters were collected. Further biases can result from cognitive impairment and possible memory distortion. Thus, the true levels of alcohol consumption at baseline and follow-up may have been underestimated. However, we used the same self-report methods that large representative studies use to estimate the consumption of alcohol, which is legal and culturally well-accepted in Germany.

Due to the analysis concept, we cannot rule out that problems of power led to several statistical results. However, we corrected the *p*-values for alpha error inflation. The LOCF method was used for imputation based on the assumption that no further changes in consumption behavior are to be expected among individuals who are no longer participating. To validate these results, a complete-cases analysis was conducted as a sensitivity analysis. This analysis confirmed the primary results. Additional strengths of the study are that we took a gender-specific approach to alcohol consumption behavior. In addition, extensive longitudinal data with different psychosocial predictor variables like depression and living situation from an MCI sample recruited throughout Germany were available for analyses.

## Conclusions

- Following the general lifestyle intervention in the 2 × 2 RCT study, people with MCI showed a reduction of their alcohol consumption after the non-specific dietary intervention.
- In particular, highly educated individuals who are still working should be the focus of counseling due to the social aspects of drinking in occupational contexts, in order to minimize the long-term negative consequences at an early stage.
- DGE recommendations should be followed to ensure maximum benefit to both genders.
- DGE recommendations seem to be easier to put into practice.
- Further research should focus in more detail on the individual aspects like cognitive level and consumption behavior prior to an intervention to provide further insights into the effects of interventions.

## Supplementary Information

Supplementary Material 1.

Supplementary Material 2.

## Data Availability

The data presented in this study are available upon reasonable request from the corresponding author. The data are not publicly available due to privacy.

## Abbreviations

CCT: Computerized cognitive training
CC: Complete case analysis
DGE: German Nutrition Society (Deutsche Gesellschaft für Ernährung)
ISCED: International Standard Classification of Education
ITT: Intention-to-treat
MCI: Mild cognitive impairment
MoCA: Montreal Cognitive Assessment
RCT: Randomized controlled trial
SDD: Standard drink
WFPB: Whole-food plant-based nutrition
