# Supplementary material for: Should I drink water or wine? Impacts of online nutritional counseling on the alcohol consumption of elderly individuals with mild cognitive impairment: Secondary Data Analysis from the RCT “BrainFit-Nutrition”
Source: BMC Public Health. 2026 Oct 3;26:2761. doi: 10.1186/s12889-026-29728-5 (PMC13633638; doi:10.1186/s12889-026-29728-5)
Supplement: Supplementary file 1 [file 12889_2026_29728_MOESM1_ESM.docx]

**Additional material 1. Sample characteristics of the complete case analysis sample (*N*=174)**

| **Variable** | **Total (*n*=174)**  ***M*(*SD*) or *n*(%)** | **Female (*n*=91)**  ***M*(*SD*) or *n*(%)** | **Male (*n*=83)**  ***M*(*SD*) oder *n*(%)** |
| --- | --- | --- | --- |
| Age, *M*(*SD*) | 70.61 (6.55) | 70.09 (0.7) | 71.19 (0.71) |
| Range | [60;86] | [60;85] | [60;86] |
| IQR | 9 | 10 | 9 |
| Sex (female), *n*(%) | 91 (52.3) | - | - |
| Education, *n*(%) |  |  |  |
| Low | 7 (4.0) | 6 (6.6) | 1 (1.2) |
| Medium | 62 (35.6) | 46 (50.5) | 16 (19.3) |
| High | 105 (60.3) | 39 (42.9) | 66 (79.5) |
| BMI, *M*(*SD*) | 25.70 (4.28) | 25.03 (4.22) | 26.44 (4.26) |
| Employment (yes), *n*(%) | 51 (29.3) | 28 (30.8) | 23 (27.7) |
| Adherence nutrition counseling, *M*(*SD*) | 8.18 (3.11) | 7.81 (3.37) | 8.58 (2.78) |
| Cognition |  |  |  |
| MoCA t0, *M*(*SD*) | 22.36 (1.57) | 22.73 (1.33) | 21.96 (1.72) |
| Range t0 | [16;24] | [19;24] | [16;24] |
| MoCA t12, *M*(*SD*) | 24.37(2.7) | 24.76 (2.79) | 23.95 (2.55) |
| Range t12 | [16;29] | [16;29] | [16;28] |
| Alcohol consumption level (drinks/day), *M*(*SD*) |  |  |  |
| t0 | 1.62 (0.97) | 1.53 (0.84) | 1.72 (1.08) |
| Range t0 | [0.125;5.0] | [0.125;5.0] | [0.125;5.0] |
| t12 | 1.27 (0.84) | 1.28 (0.82) | 1.26 (0.88) |
| Range t12 | [0.0;5.0] | [0.0;5.0] | [0.0;4.0] |

*Notes*. Education according to ISCED (2011); BMI = Body Mass Index; t0=baseline; t12=one year follow-up
