# Supplementary material for: Should I drink water or wine? Impacts of online nutritional counseling on the alcohol consumption of elderly individuals with mild cognitive impairment: Secondary Data Analysis from the RCT “BrainFit-Nutrition”
Source: BMC Public Health. 2026 Oct 3;26:2761. doi: 10.1186/s12889-026-29728-5 (PMC13633638; doi:10.1186/s12889-026-29728-5)
Supplement: Supplementary file 2 [file 12889_2026_29728_MOESM2_ESM.docx]

# **Additional material 2. Gender analysis of the complete case analysis sample**

## Total CC-Sample

##### Changes in alcohol consumption level over time

A t-test for dependent samples was conducted to evaluate the change in daily consumption within the groups. There was one extreme outlier in risky consumption at t12 (WFPB), it was checked and detected as a true outlier, and therefore remained in the data set. Thus, the assumptions were fulfilled. At t12 (*M*=1.25, *SD*=0.80), alcohol consumption in the WFPB group (*n*=82) was significantly lower than at t0 (*M*=1.53, *SD*=0.79), *t*(81)=2.99, *p*=.004, Cohen's *d*=.33 with an average reduction of ^3^⁄_10_ SDD/day. Also, in the DGE group (*n*=92), alcohol consumption at t12 (*M*=1.29, *SD*=0.88) was significantly lower than at t0 (*M*=1.69, *SD*=1.10), *t*(91)=3.69, *p*=.003, Cohen's *d*=.39. In average participants of this group reduced their daily alcohol intake by ⅖ SDD.

##### Group differences between WFPB and DGE nutritional counseling

There were no significant group differences between the WFPB group (*M*=‑0.41, *SD*=1.06) and the DGE group (*M*=-0.28, *SD*=0.86) in the reduction of alcohol consumption (*t*(170.4)=-0.865, *p*=.212). The test assumptions were fulfilled.

Multivariate analysis

Bivariately, the t12 alcohol consumption level showed relevant associations with baseline alcohol consumption level (*r*=.432, *p*<.001), with age (*r*=-.187, *p*=.013), and with the cognitive status at baseline (*r*=‑.150, *p*=.048). Therefore, these variables were also included in the regression model.

Based on the results of the assumption test, three outliers were excluded as at least two criteria of the tested outlier norms were met. All other requirements for multiple linear regression were met. The multiple linear regression (table A) resulted in a significant model, *F*(7,163)=5.762, *p*=.003, with 19.8% explanation of variance (*R^2^* = moderate). One significant variable, baseline alcohol consumption (β=.389, *p*<.001) resulted. As in the ITT sample, higher baseline alcohol consumption was associated with higher alcohol consumption 12 months later. Further, education showed a statistical trend, indicating a higher education might be associated with a higher consumption level one year later.

**Table A.** Multiple linear regression CC sample (*n*=171)

| **Variable** | ***B*** | ***SE*** | **β** | ***t*** | ***p*** | **95%-*CI* (*B*)** |
| --- | --- | --- | --- | --- | --- | --- |
| Constant | .305 | 1.184 |  | .257 | .797 | [-2.034;2.644] |
| Adherence | -.015 | .018 | -.059 | -.829 | .408 | [-.050;.020] |
| Group^§^ (WFPB) | .012 | .109 | .008 | .114 | .910 | [-.203;.228] |
| Gender~ (female) | .027 | .122 | .018 | .222 | .824 | [-.214;.268] |
| Age | -.010 | .009 | -.088 | -1.176 | .241 | [-.027;.007] |
| Education° | .215 | .119 | .138 | 1.799 | .*074* | [-.021;.451] |
| Baseline alcohol consumption | .311 | .059 | .389 | 5.313 | **<.001** | [.195;.427] |
| MoCA baseline score | .050 | .038 | .099 | 1.326 | .187 | [-.025;.125] |

*Note*. MoCA = Montreal Cognitive Assessment; ^§^nutrition counseling group (WFPB (1) vs. DGE (0)), ~gender (male (0)/female (1)). °Education was dichotomized based on ISCED classification (high education (1) vs. low education (0)). *B*: unstandardized regression coefficient; *SE*: standard error of unstandardized regression coefficient *B*; β: standardized regression coefficient; *t*: *t*-Test; *p*: Significance; 95%-CI: confidence interval of the unstandardized coefficient.

## Female CC-Sample

### Changes in alcohol consumption level over time

The female WFPB group (*n*=40) reduced for about ⅙ SDD, but the decrease from t12 (*M*=1.35, *SD*=0.78) to t0 (*M*=1.51, *SD*=0.84), *t*(39)=1.11, *p*=.137, was not significant. However, in the DGE group women (*n*=51) reduced their consumption level for about ⅓ SDD. This decrease from t12 (*M*=1.22, *SD*=0.85) to t0 (*M*=1.54, *SD*=0.85), *t*(50)=2.48, *p*=.014, Cohen's *d*=.35, was significant. Assumptions for both t-tests were fulfilled.

### Group differences between WFPB and DGE nutritional counseling

The decrease in the female DGE group was slightly bigger than in the WFPB group. Nevertheless, there were no significant group differences between the WFPB group (*M*=-0.15, *SD*=0.88) and the DGE group (*M*=-0.33, *SD*=0.94) in the reduction of alcohol consumption (*t*(89)=-0.89, *p*=.212). The requirements for the test were met.

### Multivariate Analysis

In the bivariate analysis of potentially relevant variables with the daily consumption level was a significant bivariate correlation between t12 alcohol consumption level and baseline consumption level (*r*=.397, *p*<.001) and age (*r*=-.241, *p*=.021), so this variable was also included in the regression model.

In order to carry out the multiple linear analysis, the assumptions were checked first. Apart from the outlier criterion, all other mandatory conditions for the analysis were met. As a result, two outliers were excluded due to at least two criteria were met. The multiple linear regression (Table B) resulted in a significant model, *F*(5,83)=2.523, *p*=.044, with one significant variable, baseline alcohol consumption (β=.234, *p*=.031). However, the kind of nutrition counseling and education is not significantly related to the level of subsequent consumption. Women who were in the younger age group at the start of the study appeared to have higher consumption levels at t12, corresponding to the statistical trend.. Explanation of variance (*R^2^*=132) was middle according to Cohen (1988).

**Table B.** Multiple linear regression female CC sample (*n*=89)

| **Variable** | ***B*** | ***SE*** | **β** | ***t*** | ***p*** | **95%-*CI* (*B*)** |
| --- | --- | --- | --- | --- | --- | --- |
| Constant | 2.355 | .808 |  | 2.914 | **.005** | [.748;3.962] |
| Adherence | -.007 | .020 | -.034 | -.333 | .740 | [-.047;.034] |
| Group (WFPB) ^§^ | .105 | .138 | .080 | .764 | .447 | [-.169;.379] |
| Education° | .027 | .137 | .021 | .201 | .841 | [-.244;.299] |
| Age | -.021 | .010 | -.209 | -1.962 | *.053* | [-.041;.000] |
| Baseline alcohol consumption | .190 | .087 | .234 | 2.192 | **.031** | [-.018;.363] |

*Note*. ^§^Nutrition counseling group (WFPB (1) vs. DGE (0)), ° education was dichotomized based on ISCED classification (high education (1) vs. low education (0)). *B*: unstandardized regression coefficient; *SE*: standard error of unstandardized regression coefficient *B*; β: standardized regression coefficient; *t*: *t*-Test; *p*: Significance; 95%-CI: confidence interval of the unstandardized coefficient.

## Male CC-Sample

### Changes in alcohol consumption level over time

Male participants in the WFPB group (*n*=40) reduced their alcohol consumption for about ⅖ SDD. At t12 (*M*=1.15, *SD*=0.82), their alcohol consumption was significantly lower than at t0 (*M*=1.55, *SD*=0.74), *t*(41)=3.18, *p*=.003, Cohen's *d*=.49. However, in the DGE group (*n*=41) men reduced their consumption level significantly for a ½ SDD from t12 (*M*=1.37, *SD*=0.93) to t0 (*M*=1.88, *SD*=1.34), *t*(40)=2.73, *p*=.009, Cohen's *d*=.43. Assumptions for t-tests were fulfilled.

### Group differences between WFPB and DGE nutritional counseling

The male DGE group showed a slightly higher decrease in the alcohol consumption level than the WFPB group. Nevertheless, there were no significant group differences in the reduction of alcohol consumption (*t*(70.7)=‑0.473, *p*=.319) between the WFPB group (*M*=-0.41, *SD*=0.83) and the DGE group (*M*=-0.51, *SD*=1.21). The assumptions for the test were met.

### Multivariate Analysis

Within the bivariate analysis the daily consumption level at t12 showed a significant correlation with the baseline consumption amount (*r*=.468, *p*=.002) and baseline MoCA score (*r*=.278, *p*=.011), so these variables were also included in the regression model.

The multiple linear regression (Table C), for which all assumptions were met after exclusion of one outlier, resulted in a significant model, *F*(6, 75)=5.014, *p*=.003, with one significant variable, baseline alcohol consumption (β=.454, *p*<.001) that was significantly associated with alcohol consumption 12 months later in males. Further a high education of the male participants with MCI showed a statistical trend. Neither the nutrition counseling nor the adherence or the age had any predictivity for the consumption level at t12. Explanation of variance (*R^2^*=.286) was high according to Cohen (1988).

**Table C.** Multiple linear regression male CC sample (*n*=82)

| **Variable** | ***B*** | ***SE*** | **β** | ***t*** | ***p*** | **95%-*CI* (*B*)** |
| --- | --- | --- | --- | --- | --- | --- |
| Constant | -.546 | 1.728 |  | -.316 | .753 | [-3.988;2.895 |
| Adherence | -.022 | .033 | -.069 | -.672 | .504 | [-.089;.044] |
| Group (WFPB) ^§^ | -.018 | .178 | -.010 | -.102 | .919 | [-.373;.337] |
| Age | -.003 | .014 | -.023 | -.215 | .830 | [-.032;.026] |
| Education° | .430 | .227 | .201 | 1.890 | .*063* | [-.023;.882] |
| Baseline alcohol consumption | .364 | .082 | .454 | 4.424 | **<.001** | [.200;.527] |
| MoCA baseline score | .058 | .057 | .106 | 1.022 | .310 | [-.055;.882] |

*Note*. ^§^Nutrition counseling group (WFPB (1) vs. DGE (0)), ° education was dichotomized based on ISCED classification (high education (1) vs. low education (0)). *B*: unstandardized regression coefficient; *SE*: standard error of unstandardized regression coefficient *B*; β: standardized regression coefficient; *t*: *t*-Test; *p*: Significance; 95%-CI: confidence interval of the unstandardized coefficient.
